# Potential Application of Non-Invasive Optical Imaging Methods in Orthodontic Diagnosis

**DOI:** 10.3390/jcm13040966

**Published:** 2024-02-08

**Authors:** Jae Ho Baek

**Affiliations:** F.E.S. Research Lab., Ulsan 44705, Republic of Korea; jhbaek@fes.or.kr; Tel.: +82-010-5060-0207

**Keywords:** optical coherence tomography, optical Doppler tomography, periodontal ligament, dental plaque, decalcification, early diagnosis

## Abstract

During orthodontic treatment, the early diagnosis of microscopic changes in soft and hard tissues, including periodontal tissue, is very important to prevent iatrogenic side effects like root resorption and periodontal diseases. Cervical periodontal tissue is the most critical area that reacts first to mal-habits or orthodontic forces, and it is also the place where bacteria deposits in the early stage of periodontal diseases. The early diagnosis of hard tissue changes, such as demineralization, is also very important in maintaining a patient’s health during orthodontic treatment. Many diagnostic devices, including radiographic equipment and intra-oral scanners, are helpful in diagnosing these problems, but have certain limitations in invasiveness and precision. The purpose of this study is to verify the possible utilities of non-invasive diagnostic devices in the orthodontic field that can compensate for these limitations. For this, non-invasive optical diagnostic devices, including optical coherence tomography and optical Doppler tomography, were used in vivo with animal and human examination for hard and soft tissues. These devices can provide real-time three-dimensional images at the histological scale. The results of this study verified these devices can be used in clinical practice during orthodontic treatment and introduced a new diagnostic paradigm differentiating microstructural changes in tissues in orthodontic diagnosis.

## 1. Introduction

Malocclusion can be broadly divided into problems in basal bones and in alveolar ones. Of course, problems in these two parts may become etiological factors through mutual complex effects. Excluding problems in the basal bone area and only looking at malocclusion in the alveolar bone area, malocclusion is caused when the periodontal tissue reacts to internal and/or external influences applied to the teeth, resulting in deformation of the alveolar bone. It is often referred to as tooth movement, but in reality, it is more logical to understand it from the perspective of restoring deformation of the alveolar bone. Numerous receptors within the periodontal ligament, including nociceptors and mechanoreceptors, play an important role in the mechanism by which the shape of the alveolar bone changes [1,2,3]. From an etiological perspective, when bad muscle habits exert pressure on the periodontal ligament through the teeth, the stretch receptors within the periodontal ligament, like periodontal Ruffini endings, respond to this pressure and sometimes attempt to recover [4,5], but if the pressure is continuously applied over a certain period of time, they react and induce structural changes to the alveolar bone. This brings about irreversible changes. For example, if a lower lip sucking habit continues, the maxillary anterior alveolar bone is deformed labially, causing an overall V-shape arch deformation, and the mandibular anterior alveolar bone is deformed lingually, changing into a square arch shape [6], resulting in occlusal collapse. At the starting point of all these processes is periodontal tissue, including the periodontal ligament. Even in cases where abnormal force is applied by an orthodontic device and iatrogenic damage is done to the alveolar bone, changes such as redness of the cervical gingiva occur in the periodontal tissue before the start of irreversible side effects [7]. If we could diagnose pathological changes in periodontal tissue early at a microscopic scale, we could pioneer a new orthodontic diagnostic field in two aspects. The first aspect is the early diagnosis of signs of tissue changes and malocclusion to possibly find and resolve the causal factors, and the second aspect is the early diagnosis of signs of possible iatrogenic side effects during orthodontic treatment to possibly prevent irreversible damages. To achieve this, a method is needed to objectively diagnose very detailed changes in periodontal tissue. Despite the recent development of diagnostic equipment that can evaluate periodontal tissue more precisely than before in all fields, such as oral scanners and cone-beam computed tomography (CBCT), these devices have clear limitations. Oral scanners have the disadvantage of being less sophisticated for observing minute changes in periodontal tissue and providing only surface information [8]. CBCT is not suitable for observing soft tissue changes yet and has disadvantages such as concerns about radiation exposure [9,10,11]. Diagnostic equipment that can acquire three-dimensional optical images using visible light as source, such as optical coherence tomography (OCT) [12,13] or optical Doppler tomography (ODT) [14,15], can provide non-invasive, micrometer-scale histological three-dimensional images that have been used for a long time in dermatology [16,17] and ophthalmology [18,19]. However, efforts to apply it in the dental field have been very limited [20,21], and, in particular, there have been very few attempts to utilize it in the orthodontic field [22,23]. If non-invasive optical diagnosis methods can prove to be applicable in the field of orthodontic treatment, the acquired information through this will be of great help in the early diagnosis and prevention of undesirable side effects that may occur in patients during orthodontic treatment that requires a long treatment period, especially for growing children and pregnant women who are vulnerable to radiation. This study first evaluated the possibility of using optical imaging in periodontal tissue using a white rat to set up devices for the application, and applied the protocol to human tissues, including periodontal tissues and hard tissues, in vivo. The method used here will serve as the cornerstone for applying optical imaging devices to orthodontic patients using removable or fixed appliances in the future and for establishing the standard for interpreting acquired optical images. The goal of this study is to objectively demonstrate this applicability. 

## 2. Materials and Methods

**Figure 1:**
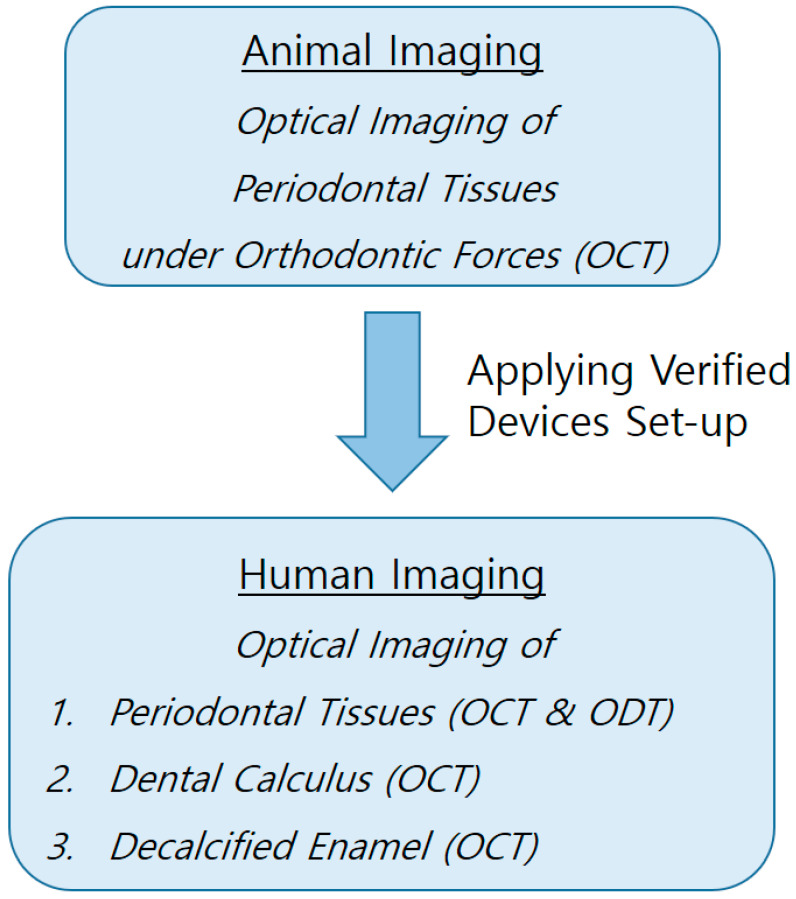
Schematic diagram of this study. It was designed to perform animal imaging first to evaluate possible problems before human application.

This experiment was designed to first conduct animal imaging to verify the usability and set up the devices, and then to apply it to the human body based on the results (Figure 1).

### 2.1. OCT and ODT

**Figure 2:**
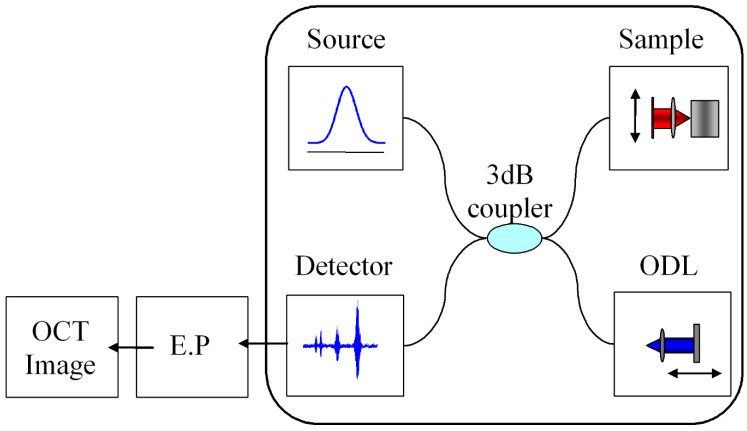
Basic OCT system. Basically, light in the visible light range is irradiated to the sample and the scattered waves are converted into a three-dimensional image. It is non-invasive and has the advantage of acquiring three-dimensional images in a short time.

A time-domain OCT and ODT system was used with a fiber-based Michelson interferometer (Figure 2) [24]. It utilized a broadband light source with an output power of 4 mW, the center wavelength was *λ*_c_ = 1310 nm, and the bandwidth was Δ*λ* = 58 nm. The detected signal from the fiber-based Michelson interferometer was band-pass filtered, demodulated electronically, sampled by a data acquisition board, and finally constructed into a two- or three-dimensional image with a personal computer. The axial and the lateral resolution were measured to be 14 µm and 10 µm in this study, respectively, which might be enough to detect changes in the periodontal ligament (which have an average 0.15 to 0.21 mm width in humans) precisely. The animal study was approved by the Ulsan University Institutional Animal Care and Use Committee (protocol code MOST M10331000001-04LO300-00110, December 2007). Ethical review and approval were waived for human imaging due to (1) non-invasive diagnostic imaging, (2) no use of drugs, (3) no tissue sample collection, (4) the use of already-proven equipment, and (5) no collection of private patient data. 

### 2.2. Animal Imaging

**Figure 3:**
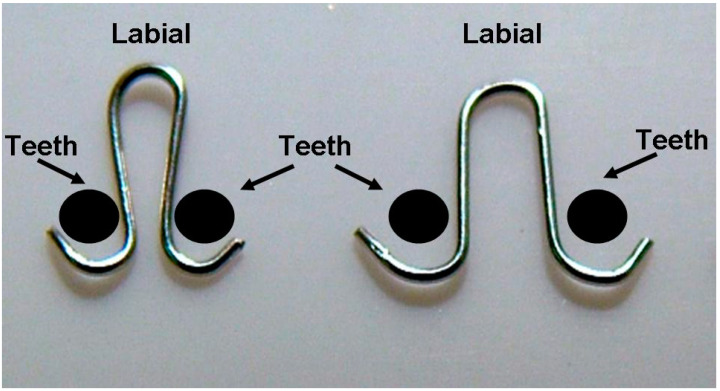
Spring design for applying orthodontic force. After pre-activation (**right**), the spring was positioned between the mandibular anterior teeth of the rat.

**Figure 4:**
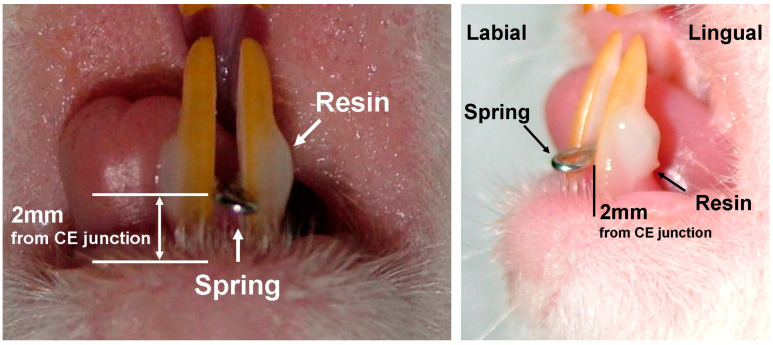
Spring positioning. The spring was positioned 2 mm above the CEJ using light cured orthodontic resin.

A 10-week-old white rat (Sprague Dawley, male) was selected. Light lateral orthodontic force (10 gm) was applied on the mandibular incisors. For this, an individualized round loop spring (Elgiloy (0.018 inch), RMO, Denver, CO, USA) was designed. After pre-activation of the control force, the spring was heat-treated to ensure continuous light force during the experiment (Figure 3). All of the processes in this study were conducted under the guide of the institutional animal care and use committee at Ulsan University (Ulsan, Republic of Korea). General anesthesia was administered by the injection of tribromo-ethanol (2,3,4-tribromo-ethanol, 300 mg/kg) via IM following ether inhalation [25]. Under general anesthesia, the prepared springs were inserted between the mandibular incisors. To reinforce retention, light cured resin (Light Bond, Reliance Ortho Prod, Itasca, IL, USA) was added over both ends of the spring following sequential processes including tooth surface cleaning with pumice and a micro-brush connected to a low-speed micro-engine, acid etching (37% phosphoric acid) on the lateral surfaces of the mandibular incisors for 15 s, washing with saline for 10 s, and complete drying with dehydrated air. The lateral ends of the springs were positioned 2 mm above the cementoenamel junctions (CEJ) to avoid spring distortion by rapid tooth eruption and/or irritation during mastication (Figure 4). The rat was cared for in a cage for 5 days. Routine checks about the amount of daily food consumption and spring retention were performed every day to reduce the animal’s possible discomfort and maintain the stability of the applied forces. After 5 days, OCT imaging evaluated periodontal ligaments around the mandibular incisors of the rat. The target area for imaging was the periodontal ligament and ligament space 2 mm beneath the CEJ, which is the easiest area to be examined with OCT in real clinical situations and can show the most critical responses of periodontal ligaments in the cervical area under orthodontic forces. The width of the periodontal ligament was measured. The animal experiment was designed as a preliminary one before applying the optical imaging devices to human oral tissues. Through this, the setup of devices was checked, and the effects of factors that can cause unnecessary noise, such as saliva, were evaluated. 

### 2.3. Human Imaging

**Figure 5:**
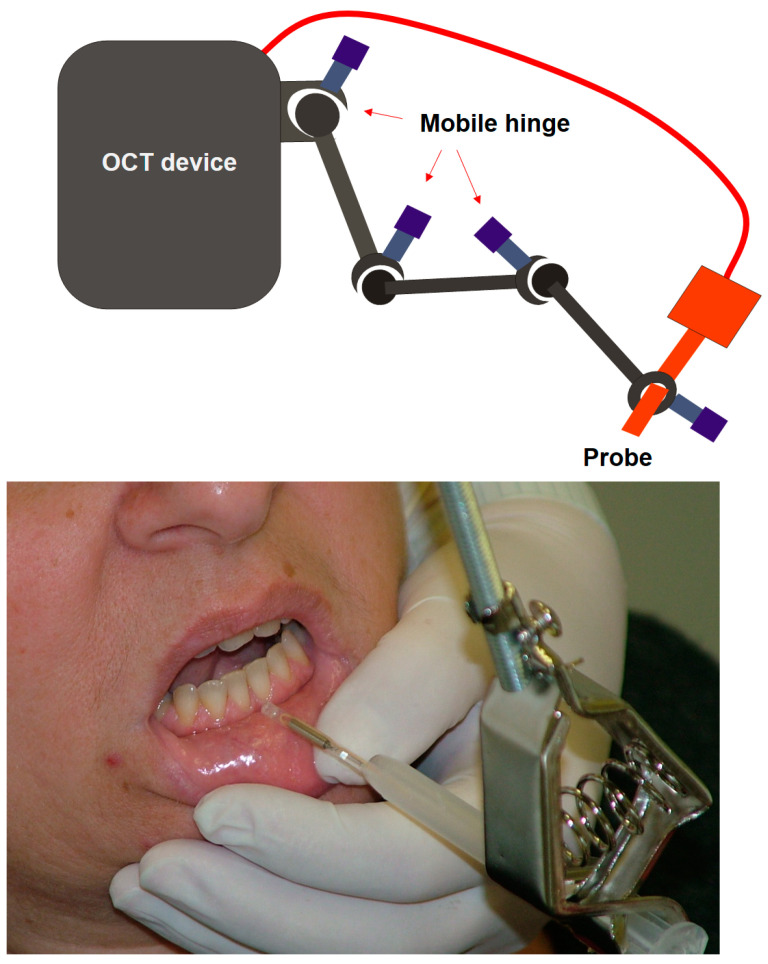
OCT probe for human imaging. To minimize the noise from the movement of the operator and patient, a specially designed probe holding device was used in this study.

A 46-year-old female without brackets was selected for optical imaging. The verified devices used in the animal experiment with the same setup were used. The reason for selecting a patient without orthodontic brackets is because this study was designed as a preliminary study to evaluate the possibilities and unexpected problems of optical diagnostic devices before applying them to patients with removable or fixed appliances during orthodontic treatment. All processes followed the guidelines of the Ulsan University hospital Institutional Review Board. Although a method proven in an animal experiment was used, there are still areas that need to be improved for actual clinical application. For this reason, in this study, a volunteer was selected from among the dental staff. Because OCT and ODT use light as the source, the shape of the probe can be greatly simplified, making it easy to apply in the oral cavity. However, there are some things to consider in order to actually apply optical imaging devices to humans, such as the fact that noise can occur even in delicate movement [26]. This movement includes that of both the patient and operator, and in this study, a specially designed holding device was developed and used to minimize the operator factor. This holding device can effectively reduce noise caused by the operator’s tremor (Figure 5).

## 3. Results

### 3.1. OCT Imaging of Periodontal Ligament under Orthodontic Forces

**Figure 6:**
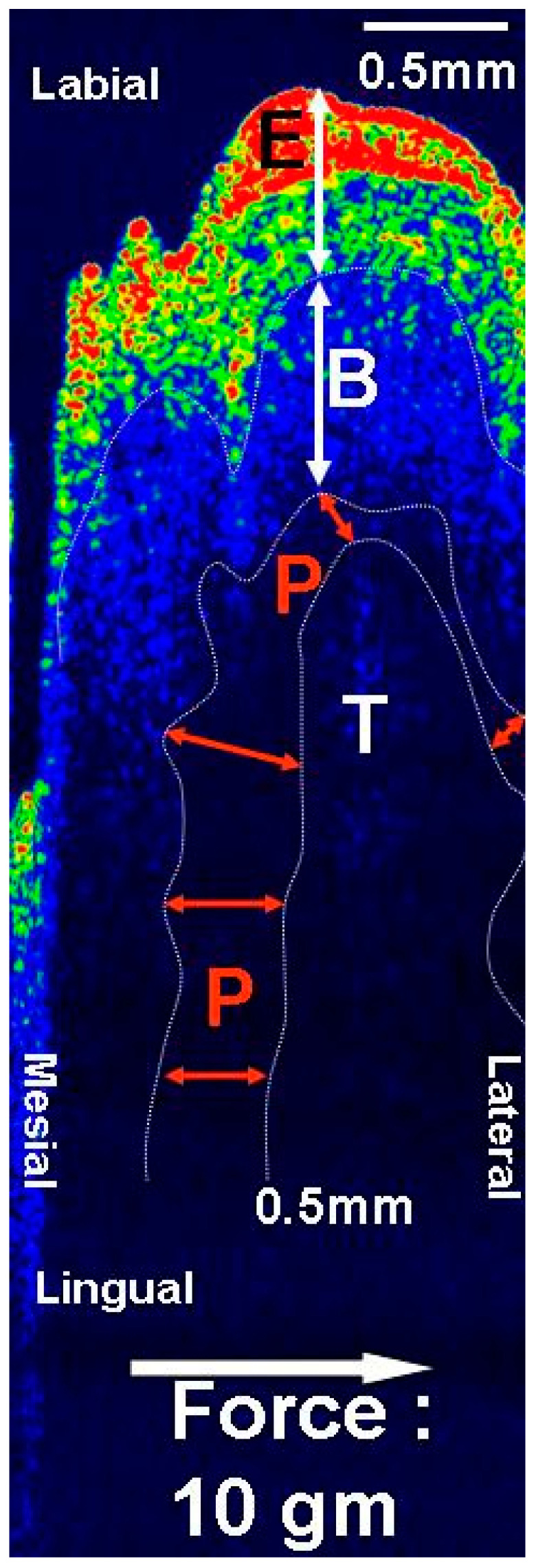
OCT image of periodontal ligament (after color mapping) under orthodontic force. E: gingival epithelium, B: alveolar bone, P: periodontal tissue, T: tooth. Enlarged periodontal ligament on tensile side and compressed widths on opposite side can be clearly distinguished.

In OCT images, the changes in widths of the periodontal ligaments can be measured from all directions, including compression and tension areas (Figure 6). 

### 3.2. OCT and ODT Imaging of Periodontal Tissues in Humans

**Figure 7:**
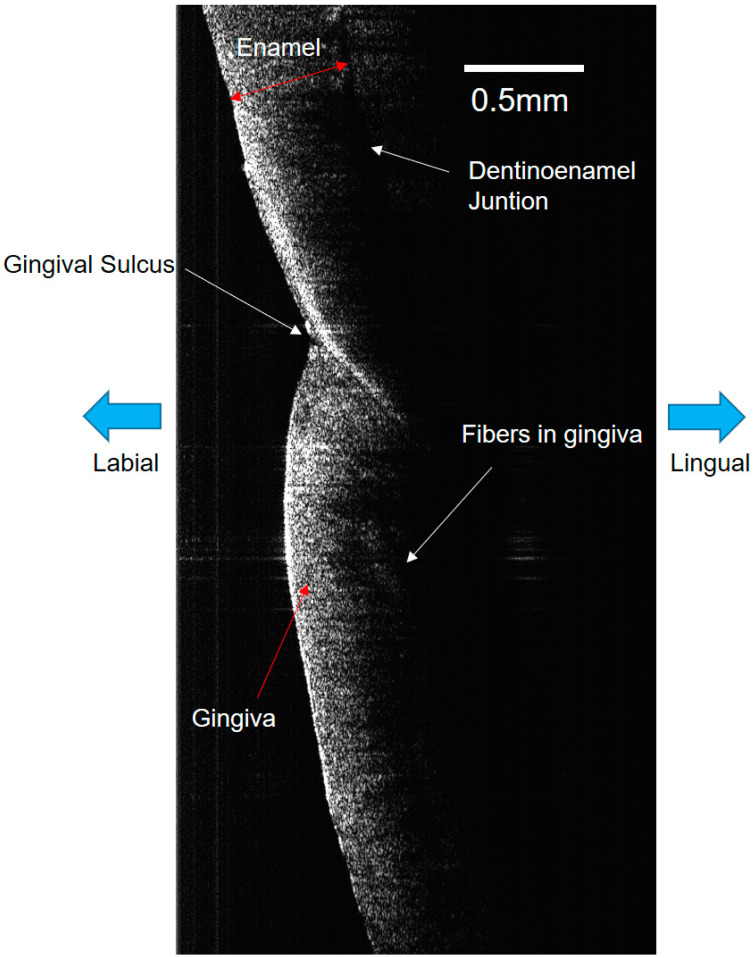
OCT image of periodontal tissue of human mandibular anterior tooth. Cervical periodontal tissue, including gingival sulcus area, which shows initial response under orthodontic forces, can be clearly identified.

**Figure 8:**
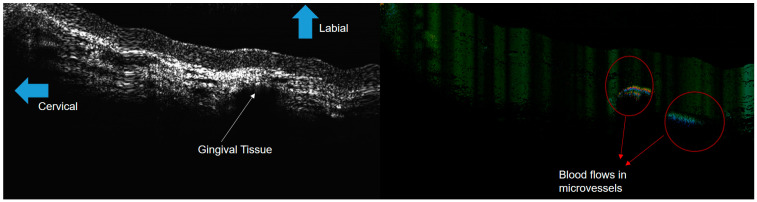
OCT (**left**) and ODT (**right**) images of human gingiva. OCT is useful for structural analysis, and OCT is adequate for analysis of flows, such as changes in blood vessels.

Effective optical imaging of periodontal tissues in the mandibular anterior region could be acquired in vivo (Figure 7). In particular, the distribution of micro-vessels within the periodontal tissue, which are difficult to identify with other existing diagnostic devices, were able to be distinguished by ODT. While OCT is efficient for structural analysis, ODT has the advantage of being able to distinguish minute changes in blood flow within blood vessels (Figure 8) [14]. 

### 3.3. OCT Imaging of Dental Plaque and Decalcified Area 

**Figure 9:**
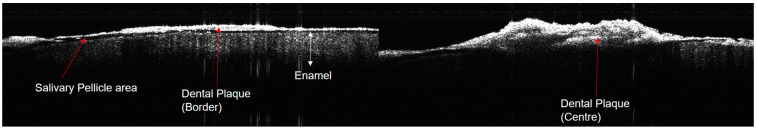
An in vivo OCT image of dental plaque on a mandibular anterior tooth. The salivary pellicle layer is clear at the border area of dental plaque (**left**) but obscure in the center (**right**). Several laminated layers just like a ‘tree ring’ show the sequential growth of dental plaque (right).

**Figure 10:**
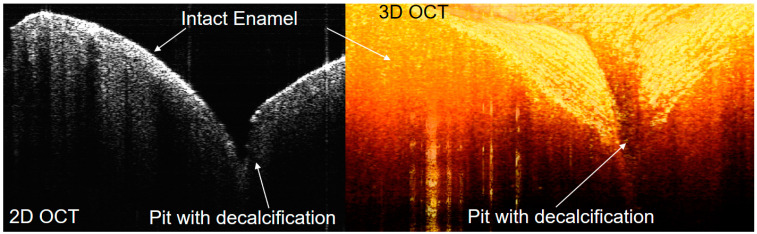
Incipient caries on occlusal enamel of mandibular molar. OCT and ODT images can be acquired as 2D or 3D. Precise diagnosis for initial decalcification can be performed more accurately in 2D images, although 3D imaging is more useful for general inspection.

Optical imaging of dental plaque on the surface of a mandibular anterior tooth in vivo was also acquired in this study. The salivary pellicle layer, which was clear at the margin of dental plaque, became obscure toward the center area, showing that there was a close correlation between the growth of dental plaque and the soundness of the salivary pellicle [27]. In addition, the lamination layers according to the growth of dental plaque were identified (Figure 9). Initial demineralization on the enamel, which was difficult to clearly distinguish clinically and/or radiologically, could also be diagnosed using OCT (Figure 10) [28].

## 4. Discussion

Optical imaging is a non-invasive diagnostic method that has been used in various medical fields, even including cancer diagnosis recently [29,30,31], but it has several limitations in dental applications [32]. The first problem is noise caused by movement of the patient and/or the operator, and scattered waves from the saliva are ball and chain [20]. However, these problems can be solved by artificial intelligence (A.I.) using big data like in other diagnostic devices, including magnetic resonance imaging (MRI) [33,34]. The second problem is the limited penetration depth in intra-oral soft tissues [35]. One of the reasons optical imaging has been widely applied, especially in the ophthalmic area, is because our eyes have a deep light penetration range [36]. In dentistry, in the case of periodontal tissue, it is technically difficult to further improve the penetration depth due to the distribution of fibrous tissues and many blood vessels which block light transmission [21]. However, the results of this study showed that the current range is sufficient for diagnosing minute changes in periodontal tissues in the cervical region, which shows the earliest response during orthodontic tooth movement [20,37]. In addition, since the cervical area is the most sensitive area to various pathologic changes that may occur during orthodontic treatment, including the deposition of dental plaque, we will be able to make more accurate diagnoses by adding microscopic information in this area [38]. In this study, it was found through animal experiments that optical imaging is useful to diagnose microscopic changes in periodontal tissues under orthodontic force, and it was also confirmed that these results can be applied to humans in vivo. Since the methods used in this study allow for the real-time, non-invasive evaluation of the response of actual periodontal tissues under orthodontic forces during real patients’ treatment, the need for indirect evaluations through animal experiments or finite element analysis can also be reduced while increasing the accuracy of evaluations [39]. The results of this study showed the early diagnosis of initial decalcification and the growth of dental plaque is possible using optical imaging. Demineralization and periodontal diseases are among the iatrogenic side effects that commonly occur during orthodontic treatment. Non-invasive diagnosis using optical imaging will enable a new approach to research on not only the prevention of demineralization but also finding remineralization materials and methods [40,41,42]. For example, the clinical effects of various substances other than those already known, including fluorine, could be effectively evaluated through optical imaging. Optical imaging of plaque development can lead to new approaches to orthodontic device surface treatment and structural design by assessing the distribution of plaque around orthodontic appliances. It may open the possibility of conducting systematic clinical research to find clues to prevent periodontal disease not only in orthodontic patients but also in patients who do not undergo orthodontic treatment. The measurement of blood flow changes by ODT can supplement information that cannot be diagnosed in structural changes by OCT [43]. For example, by diagnosing blood flow changes when an inflammatory reaction occurs due to orthodontic force or diseases, information on periodontal tissue damage or recovery can be identified earlier before structural changes occur. Changes in blood flow can occur in both normal orthodontic tissue remodeling and pathological inflammation [44,45]. So, this is one of the future tasks that needs to be further established to differentiate these two reactions. It will be necessary to evaluate the amount and distribution of blood flow changes using ODT and the structural changes of alveolar bone using OCT for this. By evaluating the correlation between these data and existing clinical evaluation indicators, including the bleeding index and pocket depth [46,47], it will be possible to establish standards for differential diagnosis. The advantages that we can gain by including not only teeth but also periodontal tissue changes in the field of orthodontic diagnosis using non-invasive optical imaging at the histological scale are endless, excluding the points mentioned above. There are some technical issues that need to be resolved in order to actively utilize optical imaging diagnostic devices in actual clinical dental practice, including orthodontics, but it is not impossible now. If OCT or ODT is combined with the currently popular oral scanner, the burden of additional training for operators and introduction costs can be reduced. In addition, the previously mentioned noise problem can be solved by A.I. using training through a lot of clinical information, as seen in the development progress of facial recognition technology. Just as our understanding of the universe changed significantly before and after the Hubble Space Telescope, I believe that optical imaging diagnosis can change our perspective in the area of orthodontic diagnosis. 

## 5. Conclusions

Optical diagnostic imaging devices have sufficient objective potential for the diagnosis of soft tissues, including periodontal tissue and hard tissue, in the orthodontic field. In addition to solving some technical problems, the results of this study open a new horizon in the field of orthodontic diagnosis through ongoing research to understand the correlation between changes in periodontal tissue and tooth movements during orthodontic treatment or pathologic progresses. Through this study, it was found that OCT and ODT can provide two-dimensional and/or three-dimensional images of periodontal and hard tissue changes during orthodontic treatment at the histological level non-invasively in both animal experiments and humans in vivo. Specifically, it was found that these devices can be used during orthodontic treatment in various areas, such as estimating microscopic changes in periodontal tissue, which are very important in evaluating biomechanical effects, the early diagnosis of periodontal diseases, and enamel demineralization. But, it is a task that must be solved in the future to collect more data by applying these devices to orthodontic patients with fixed or removable appliances and then verifying the correlation between the results and existing information to establish standards for the objective analysis of the data.

## Data Availability

No new data were created or analyzed in this study. Data sharing is not applicable to this article.
